# Production of IL-16 correlates with CD4+ Th1 inflammation and phosphorylation of axonal cytoskeleton in multiple sclerosis lesions

**DOI:** 10.1186/1742-2094-3-13

**Published:** 2006-05-26

**Authors:** Dusanka S Skundric, Juan Cai, William W Cruikshank, Djordje Gveric

**Affiliations:** 1 Department of Neurology, Wayne State University School of Medicine, Detroit, MI 48201, USA; 2 Department of Immunology and Microbiology, Wayne State University School of Medicine, Detroit, MI 48201, USA; 3 Pulmonary Center Boston University School of Medicine, Boston, MA 02118, USA; 4 Department of Neuroinflammation, Institute of Neurology, University College London WC1N 1PJ, UK

## Abstract

**Background:**

Multiple sclerosis (MS) is a central nervous system-specific autoimmune, demyelinating and neurodegenerative disease. Infiltration of lesions by autoaggressive, myelin-specific CD4+Th1 cells correlates with clinical manifestations of disease. The cytokine IL-16 is a CD4+ T cell-specific chemoattractant that is biased towards CD4+ Th1 cells. IL-16 precursor is constitutively expressed in lymphocytes and during CD4+ T cell activation; active caspase-3 cleaves and releases C-terminal bioactive IL-16. Previously, we used an animal model of MS to demonstrate an important role for IL-16 in regulation of autoimmune inflammation and subsequent axonal damage. This role of IL-16 in MS is largely unexplored. Here we examine the regulation of IL-16 in relation to CD4+ Th1 infiltration and inflammation-related changes of axonal cytoskeleton in MS lesions.

**Methods:**

We measured relative levels of IL-16, active caspase-3, T-bet, Stat-1 (Tyr ^701^), and phosphorylated NF(M+H), in brain and spinal cord lesions from MS autopsies, using western blot analysis. We examined samples from 39 MS cases, which included acute, subacute and chronic lesions, as well as adjacent, normal-appearing white and grey matter. All samples were taken from patients with relapsing remitting clinical disease. We employed two-color immunostaining and confocal microscopy to identify phenotypes of IL-16-containing cells in frozen tissue sections from MS lesions.

**Results:**

We found markedly increased levels of pro- and secreted IL-16 (80 kD and 22 kD, respectively) in MS lesions compared to controls. Levels of IL-16 peaked in acute, diminished in subacute, and were elevated again in chronic active lesions. Compared to lesions, lower but still appreciable IL-6 levels were measured in normal-appearing white matter adjacent to active lesions. Levels of IL-16 corresponded to increases in active-caspase-3, T-bet and phosphorylated Stat-1. In MS lesions, we readily observed IL-16 immunoreactivity confined to infiltrating CD3+, T-bet+ and active caspase-3+ mononuclear cells.

**Conclusion:**

We present evidence suggesting that IL-16 production occurs in MS lesions. We show correlations between increased levels of secreted IL-16, CD4+ Th1 cell inflammation, and phosphorylation of axonal cytoskeleton in MS lesions. Overall, the data suggest a possible role for IL-16 in regulation of inflammation and of subsequent changes in the axonal cytoskeleton in MS.

## Background

Multiple sclerosis (MS) is an inflammatory, demyelinating and neurodegenerative disease of central nervous system (CNS) [1,2]. The complex immunopathology of MS is initiated by infiltration of macrophages and lymphocytes into brain and spinal cord [3]. In patients with MS, magnetic resonance imaging (MRI) has confirmed that intrathecal infiltration correlates with clinically active, acute, and relapsing stages of disease. Infiltrating immune cells – comprised of myelin-specific and nonspecific autoaggressive and regulatory T cells, B cells, NK, NK-T and dendritic cells – are essential for myelin stripping, degeneration of axonal cytoskeleton, and/or damage to oligodendrocytes in MS lesions [4]. Based on a gradually decreasing extent of inflammation over time, MS lesions are typically classified as acute, subacute and chronic [5]. In multifaceted interactions between infiltrating cells, and between infiltrating cells and local glial cells and/or axons, a CD4+ Th1 cell subset has an exceptional role because it includes potentially autoaggressive cells specific for immunodominant epitopes of myelin proteins. Regulation of Th1 immunity, which includes differentiation of naïve CD4+ T cells into IFNγ-producing Th1 cells, is tightly controlled by T-bet, a member of T-box transcription factor family. In Th1-mediated autoimmune diseases, T-bet is instrumental for generation of autoreactive CD4+ Th1 cells [6-8]. Induction of T-bet depends on signaling through the signal transducer and activator of transcription-1 (Stat-1). Activation of Stat-1 occurs through phosphorylation of either tyrosine-701 or serine-727 [9,10].

Homing of mononuclear cells, including encephalitogenic CD4+ Th1 cells, into the CNS is tightly regulated by chemoattractant factors [11]. As opposed to chemokines, which bind to chemokine-specific receptors and do not discriminate between distinct cell phenotypes, IL-16 binds to CD4 co-receptors and selectively chemoattracts CD4+ T cells [12-14]. More importantly, the chemotactic properties of this cytokine are biased towards a Th1 subset, because of the close functional relationship between CD4 molecules and CCR5 [15]. The human IL-16 precursor (pro-IL-16) is a 631-amino acid, two-PDZ domain-containing protein that is constitutively produced in unstimulated peripheral T lymphocytes. Following CD4+ T cell activation through T cell receptors (TCR) or by cytokines, active caspase-3 cleaves a 121-amino acid C-terminal portion, which is then secreted and becomes available to bind to CD4 receptors. In addition to CD4+ T cell migratory responses, IL-16 also regulates T cell activation, growth, CD25 and MHC class II expression, cytokine synthesis, and modulation of chemokine-induced chemoattraction [16,17]. Thus, IL-16 is a proinflammatory and immunoregulatory cytokine, which has an important role in recruitment and activation of CD4+ Th1 cells [18].

We previously reported a prominent role for IL-16 in immune regulation of relapsing-remitting EAE in mice, which impacted the severity of relapsing disease, of inflammation, and of demyelination, as well as the extent of axonal damage [19]. We provided evidence of regulation of IL-16 in EAE, which suggested that production of secreted IL-16 occurs within the CNS, and that IL-16 has a role in specific chemoattraction of CD4+ T cells in EAE [20]. However, the regulation of IL-16 in MS itself, and the potential significance of IL-16 in regulating specific CD4+ Th1 inflammation and subsequent tissue damage in MS remain largely unexplored.

In this study we investigated correlations between the regulation of IL-16, regulation of CD4+Th1 inflammation, and inflammation-induced changes in axonal cytoskeleton in lesions sampled from 39 autopsies of patients with MS and 19 controls. We found marked increases in IL-16 and active caspase-3 expression in lesions and in adjacent normal appearing white matter (NAWM). Similarly, specific increases in T-bet and phosphorylated Stat-1 were measured in MS lesions, providing a correlation between CD4+ Th1 inflammation and intrathecal IL-16 production. We observed T-bet+IL-16+ infiltrating cells in MS lesions. Increases in phosphorylated neurofilament medium and heavy chains [NF (M+H) P] suggested initial, inflammation-induced changes in axonal cytoskeleton in MS lesions and NAWM. We observed IL-16 adjacent to abnormal-appearing axonal cytoskeletons. Overall, our data suggest a role for IL-16 in immune regulation of CD4+ Th1-specific inflammation, and subsequent changes in axonal cytoskeleton, in MS.

## Methods

### Tissue

Postmortem snap-frozen tissue, from 39 clinically and histopathologically definite multiple sclerosis patients and 17 controls, was obtained from the Neuroresource Tissue Bank at the Institute of Neurology, London, UK. All MS cases were classified as secondary progressive (SP) with significant increasing disability and relapsing-remitting clinical course. The average age, gender, postmortem time (PM time), Expanded Disability Status Scale score (EDSS) [21], and duration of disease are presented in Table 1. Cause of death in the normal control category was unrelated to diseases of the nervous system. Multiple sclerosis lesions were classified according to Li et al. [5].

**Table 1:** Summary of clinical data

	Control	Multiple sclerosis
Number of cases	17	39
Age (years – mean ± SD, range)	59 ± 15 (34–78)	54 ± 13 (31 – 66)
Gender (F/M)	4/13	27/12
PM time	24 ± 9	21 ± 6
Duration of MS (years)	NA	22 ± 11
EDSS	NA	9
Clinical classification	NA	SP

NA = non applicable; F = female, M = male; PM = post mortem; EDSS = Expanded Disability Status Scale score; SP = secondary progressive

A total of 62 frozen blocks (0.5–1 cm^3^) of brain and spinal cord tissue were dissected. These included 13 acute (AL), 13 sub-acute (SAL), and 13 chronic (CL) MS lesions, 17 adjacent areas of macroscopically normal-appearing white (NAWM), and 6 areas of normal-appearing grey matter (NAGM). From each tissue block, 10-μm frozen sections were cut or tissue was homogenized for protein isolation.

### Western blot

Proteins were isolated from snap-frozen tissue blocks of approximately 1 g wet weight. Tissue was homogenized in Tris-HCl buffer (100 mM Tris, pH 8.1 with 1% Triton X-100) [22] by sonication. Tissue suspensions were spun at 20,000 g for 45 minutes at 4°C. Supernatants were collected and stored at -70°C until use. Protein concentrations were determined by the Lowry method. Equal amounts of protein (approximately 30 μg/lane) from each sample were loaded per lane for western blot analysis. Protein samples were loaded with reducing conditions, and resolved by electrophoresis in NuPage Bis-Tris (4–12%) gels (Invitrogen, Carrsbad, CA). Electrophoresed proteins were then transferred from the gel onto nitrocellulose membrane. The membrane was blocked, and then probed with the appropriate primary antibody overnight at 4°C, washed three times with 0.1% Tween 20- Tris-buffered saline, and than incubated with peroxidase-conjugated secondary antibody. The membrane-bound peroxidase activity was detected by using ECL Plus western blotting detection kits (Amersham, Arlington Heights, IL). Chemiluminescent images were captured and analyzed by a Kodak Digital Science Image Station 440 CF. All blots were studied within the linear range of exposure. In each sample, levels of IL-16, active caspase-3, T-bet, Stat-1 (Tyr^701^), and NF (M+H)-P, were normalized to corresponding levels of GAPDH.

### Immunostaining and confocal microscopy

Frozen sections, 10 μM thick, were used to analyze phenotypes of infiltrating cells by immunofluorescence following a routine procedure [19]. Briefly, sections were air-dried, acetone-fixed, and treated with 10% normal donkey serum for 10 minutes, followed by overnight incubation with relevant primary antibody (Table 2) in a moist chamber at +4°C. The slides were then washed and incubated with secondary fluorochrome-labeled antibodies for 30 minutes. The following secondary antibodies were used: anti-goat, anti-rabbit and anti-mouse IgG-HRP conjugated at 1:10,000, (Santa Cruz Biotechology, CA). Nuclear staining was performed using 30 nM 4',6-diamidino-2-phenylindole, dihydrochlpride (DAPI) (Molecular Probes). The slides were washed, mounted in Gelmount (Biomeda, Foster City, CA), and analyzed by light and fluorescent microscopy. Images were captured on a Nikon Eclipse 600 epifluorescent microscope with a Princeton Instruments Micromax 5 MHz cooled CCD camera.

**Table 2:** Primary antibodies used for immunostaining and western blot

Antigen	Clone	Dilution	Form	Source	Immuno/WB
IL-16	14.1	1:1000		BD Biosciences San Diego CA	WB
IL-16	14.1	1:200	PE	BD Biosciences San Diego CA	IC
G155-178	(control)	1:200	PE	BD Biosciences San Diego CA	IC
Caspase-3	Polycloal	1:1000		R&D Systems Minneapolis MN	WB
Active- Caspase-3	Polycloal	1:200		R&D Systems Minneapolis MN	IC
T-bet	Poly6235	1:500		Biolegend San Diego CA	WB
Stat-1	Polycloal	1:1000		Cell Signaling Technology Co Danvers MA	WB
Stat-6	Polycloal	1:1000		Cell Signaling Technology Co Danvers MA	WB
NF(H+M)	NP1	1:500		Chemicon Temecula CA	WB
GAPDH	Polycloal	1:1000		Santa Cruz Biotechnology Santa Cruz CA	WB
CD3	APA1/1	1:100	FITC	BD Biosciences San Diego CA	IC
CD4	RPA-T4	1:100	FITC	BD Biosciences San Diego CA	IC
CD8	3b5	1:100	FITC	Caltag Laboratories Bulingame CA	IC
CD11b/Mac-1	ICRF44	1:50	Biotin	BD Biosciences San Diego CA	IC
CD20	H147	1:500	FITC	Caltag Laboratories Bulingame CA	IC
CD83	HB15e	1:500	FITC	Caltag Laboratories Bulingame CA	IC

### Statistical analysis

All statistical analyses were done using GraphPad Prism software (GraphPad, San Diego, CA). The significance of differences between groups was calculated using Student's *t*-test. The level of statistical significance was set at *p *< 0.05.

## Results

### Levels of pro- and secreted IL-16 are distinctly regulated in acute, subacute and chronic MS lesions in brain and spinal cord

**Figure 1:**
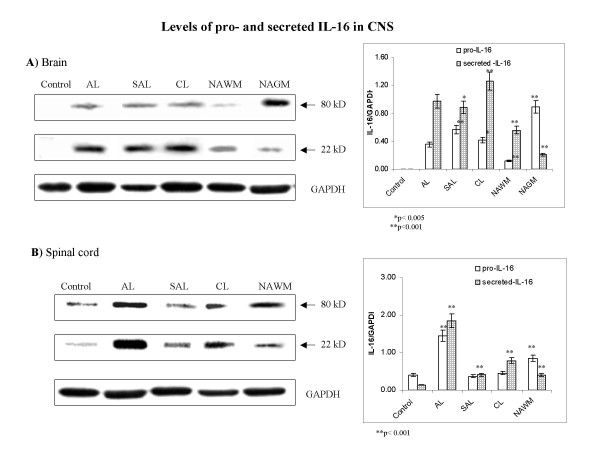
**MS lesions contain increased levels of pro- and secreted IL-16 compared to control tissue**. Markedly different relative levels of IL-16 were measured between different types of lesions and control (**A**) brain and (**B**) spinal cord tissue, by western blot. Representative blots show levels of pro-IL-16 (80 kD) and secreted IL-16 (22 kD) in: (**A**) normal control (periventricular white matter of left parietal lobe), and MS brains. Periventricular white matter of MS lesions and adjacent normal-appearing white matter were sampled from: AL (acute lesion – right parietal lobe); NAWM (normal appearing white matter – right temporal lobe); NAGM (normal-appearing grey matter – right temporal lobe); SAL (subacute lesion – right parietal lobe) and CL (chronic lesion – right parietal lobe); and (**B**) spinal cord: lumbar spinal cord was analyzed from control and MS patients. Representative blots are shown from a total of 7 experiments analyzing 39 MS cases and 19 controls. Relative levels of IL-16/GAPDH were estimated by densitometry. Mean values ± SD are shown in corresponding graphs.

MS lesions showed marked increases in levels of IL-16 precursor (pro-IL-16, 80 kD), and mature, secreted IL-16 (22 kD), compared to normal control brain and spinal cord white matter (Fig. 1). Pro-IL-16 was undetectable in control brain (Fig. 1A), and was very low in control spinal cord white matter (Fig. 1B), but was abundantly present in MS lesions in brain and spinal cord. In brain, levels of pro-IL-16 did not differ significantly between acute and chronic lesions. Interestingly, in normal-appearing white matter (NAWM) adjacent to acute lesions, pro-IL-16 levels were approximately one third those of acute lesions, while in normal-appearing grey matter (NAGM) pro-IL-16 levels were elevated more than two fold over levels in acute lesions (Fig. 1A). In MS lesions in spinal cord, levels of pro-IL-16 was greatest in acute lesions, reaching approximately five times control levels. Subacute and chronic lesions showed significantly lower levels of pro-IL-16 than did acute lesions, and these were indistinguishable from control levels. As was found for brain, spinal cord NAWM showed appreciable levels of pro-IL-16. These levels were lower than those measured in acute lesions, but still markedly higher than control levels (Fig. 1B).

Mature IL-16 showed patterns similar to those of pro-IL-16. Mature IL-16 was also undetectable in control brain white matter by western blot. An abundant presence of secreted IL-16 was measured in acute, subacute and especially in chronic MS lesions in brain. Differences between these types of lesions were not significant. NAWM and NAGM showed secreted levels of IL-16 that were approximately one half to one fifth those of acute MS lesions, but still appreciable by western blot, especially in NAWM (Fig. 1A). In normal spinal cord, secreted IL-16 was detected at very low levels. In acute spinal cord lesions, the content of secreted IL-16 was over ten times higher than basal levels. Significant downregulation of secreted IL-16 was observed in subacute and chronic lesions and in NAWM, in comparison to acute lesions (Fig. 1B). The relative levels of IL-16 in spinal cord appear to be consistently higher than those of brain.

### Regulation of active caspase-3 parallels secreted IL-16 and suggests a role for this caspase in enzymatic cleavage of pro-IL-16 in MS lesions

**Figure 2:**
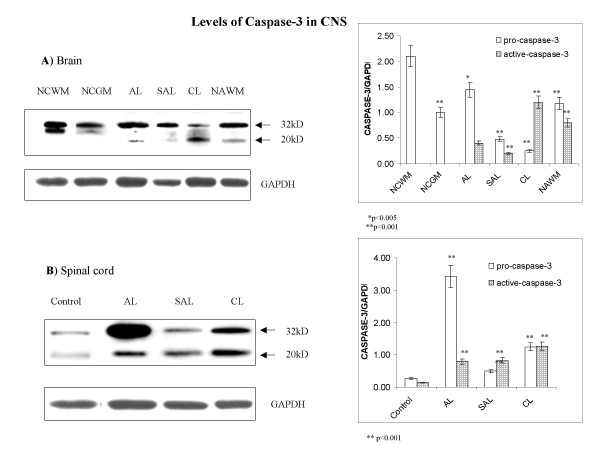
**Levels of active -caspase-3 are elevated in MS lesions**. Levels of pro-caspase-3 (32 kD) and active capsase-3 (20 kD) were measured in (**A**) brain and (**B**) spinal cord. **A**) While pro-caspase was found at relatively high levels, active-caspase-3 was not detected in normal control white matter (NCWM) and normal control grey matter (NCGM) in brain. A decrease of pro-caspase-3 was followed by an increase of active caspase-3 in MS lesions. **B**) As opposed to brain, very low basal levels of pro- and active caspse-3 were found in control spinal cord. Dramatic increases of pro-caspase-3 was measured in acute lesions (AL), which then subsided in subacute lesions (SAL) and chronic lesions (CL). Levels of active caspase-3 were markedly and persistently higher in MS lesions compared to control tissue. Representative blots are shown from a total of 5 experiments analyzing 39 MS cases and 19 controls. Relative levels of Caspase-3/GAPDH are estimated by densitometry. Mean values ± SD are shown in corresponding graphs.

We used an anti-caspase-3 antibody that reacts with both pro-caspase-3 (32 kD) and its cleavage fragment, which represents active caspase-3 (20 kD), for western blot analysis. In white and grey matter of control brains levels of pro-caspase-3 were appreciable, while active caspase-3 was not detected. In MS lesions from MS brains, levels of pro-caspase-3 were not significantly higher than basal levels but an increase in active caspase-3 was observed. (Fig. 2A). In spinal cord, control levels of pro-caspase-3 were very low but still detectable, while active caspase-3 was virtually undetectable. Compared to low basal levels in normal spinal cord, pro- and active caspase-3 were markedly increased in spinal cord MS lesions (Fig. 2B). Active caspase-3 showed high levels in chronic MS lesion of brain and spinal cord; a pattern similar to that seen for IL-16 (Fig. 1).

**Figure 4:**
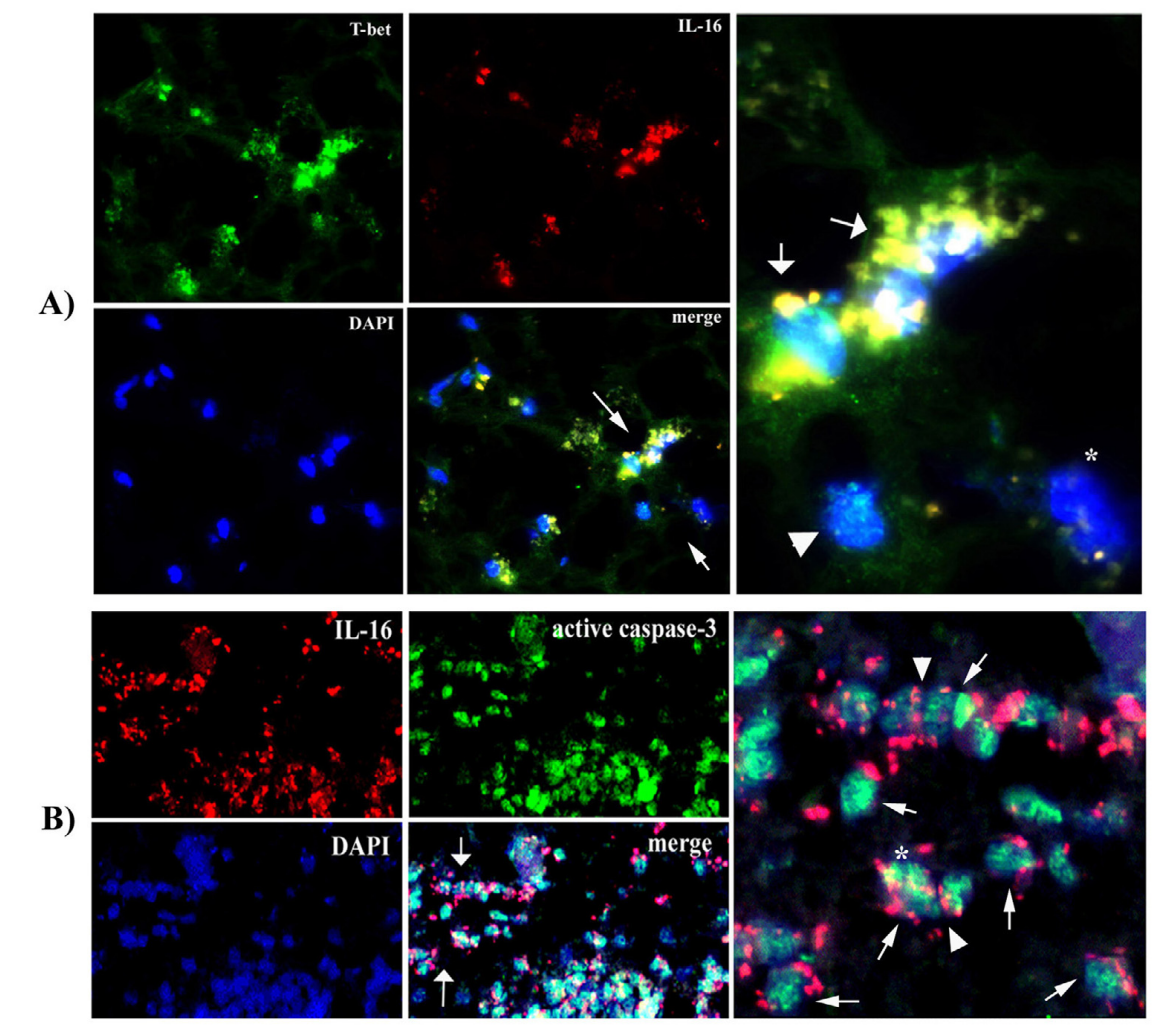
**IL-16 immunoreactivity is observed confined to T-bet+ cells and active caspase-3+ cells in MS lesions**. **A**) In acute lesions (AL) in MS brain, prominent T-bet+ cells were found in small infiltrates scattered throughout the white matter of parietal lobe. IL-16 immunoreactivity was observed at similar locations within the lesion. DAPI staining indicates that the immunostaining is localized to mononuclear cells. The merged image shows that IL-16 immunoreactivity co-localizes to some T-bet+ cells. The image on the far right shows enlarged detail from the merged image (at arrows), where IL-16 confined to T-bet+ cells (at arrows) can be better appreciated. It also shows a T-bet+ cell, which is IL-16- (arrowhead), and a T-bet-, IL-16- cell (asterisk). **B**) In an AL of lumbar spinal cord, intense immunostaining for IL-16 and for active caspase-3 is seen in dense perivenular mononuclear cell infiltrates in the white matter. A detail from the merged image (at arrows) is shown in the far right image. Most infiltrating cells exhibit a nuclear pattern of immunostaining for active caspase-3. Some of these active caspse-3+ cells are IL-16+ (at arrows). Note the distinct patterns of IL-16 and active caspase-3 immunostaining. While IL-16 nuclear immunostaining is observed occasionally (asterisk), more often it appears as cytoplasmic or membrane-bound immunostaining, or as secreted product adjacent to cells (at arrows). IL-16 was found frequently between adjoining mononuclear cells (arrowhead). Two color fluorescence × 60.

**Figure 6:**
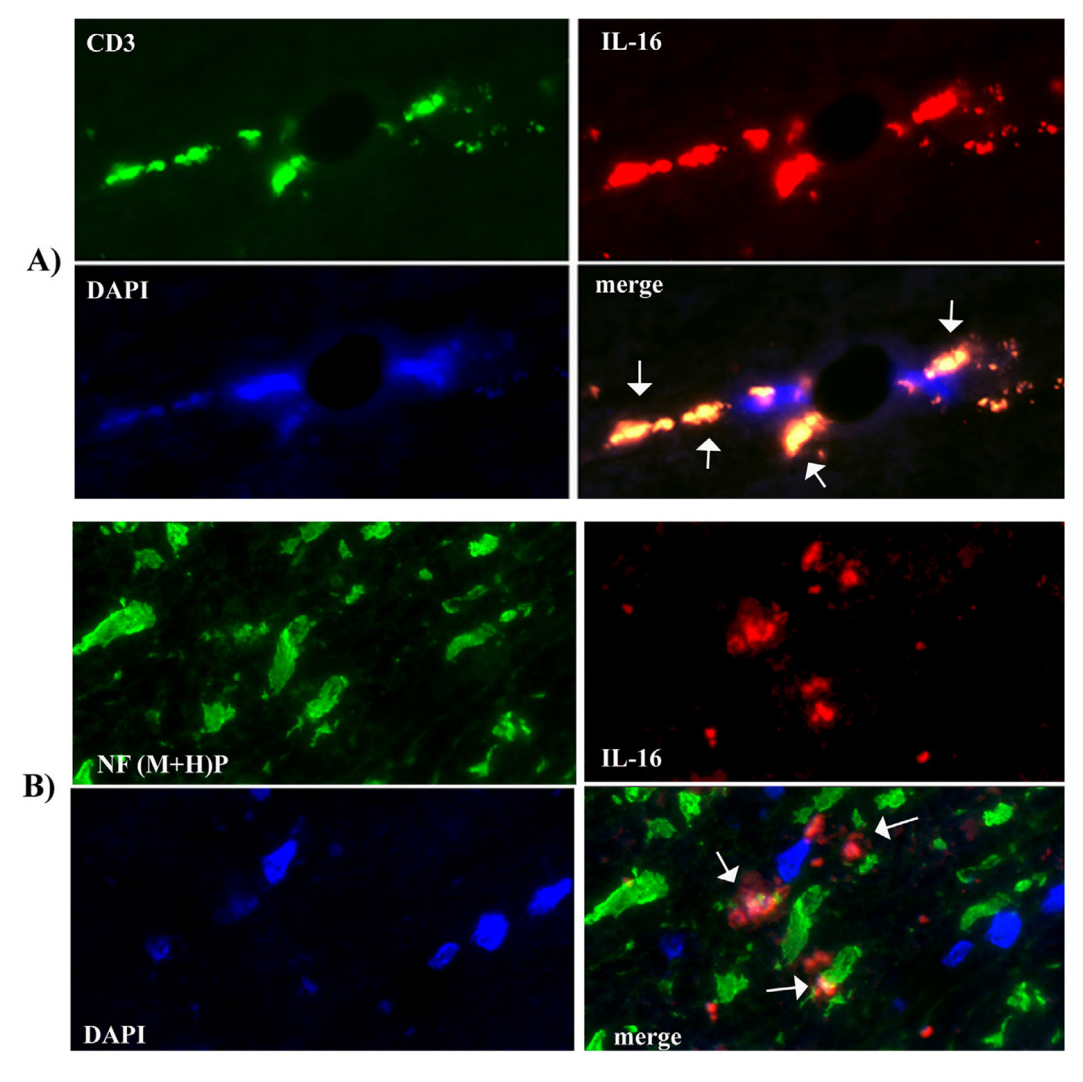
**IL-16 immunoreactivity is present in occasional infiltrating lymphocytes adjacent to degenerate axonal cytoskeletons, in normal-appearing white matter (NAWM)**. **A**) Occasional IL-16-positive CD3+ T cells were observed around small venules in NAWM adjacent to acute lesions in spinal cord (at arrows). **B**) Note degenerate axonal neurofilaments [NF(M+H)P], which appear rounded, ballooned and irregularly shaped (at arrows). Some IL-16 immunoreactivity was observed in the proximity of axons, either confined to or adjacent to sparse infiltrating mononuclear cells (merged image, arrows). Note that IL-16 does not co-localize with axonal neurofilament. Two color fluorescence × 40.

The antibody specific for IL-16 that we used for both immunostaining and western blot binds to the C-terminal portion of both pro- and secreted IL-16 and therefore does not allow distinction between two forms of IL-16 based on immunostaining. To examine whether cleavage of pro-IL-16 may occur in infiltrating IL-16+ cells, we performed double immunostaining using an antibody that recognizes the p17 subunit of active caspase-3 (Table 2) and the IL-16-specific antibody. Isotype-matched control antibody (Table 2) was used to confirm the specificity of IL-16 immunostaining (not shown). We observed numerous IL-16+ active-caspase-3+ mononuclear cells, suggesting that production of secreted IL-16 occurs within MS lesions (Fig. 4B). While active caspase-3 was confined to nuclei, IL-16 immunoreactivity was rarely observed in nuclei. This was more often found polarized on cell membranes or adjacent to mononuclear cells (Fig. 4A and 4B, and Fig. 6B). In perivenular and white matter-scattered infiltrates within MS tissus, IL-16 immunoreactivity was often found at the sites of cell-cell contact between mononuclear cells (Fig. 4A, B). In NAWM, IL-16 was observed in sparse infiltrating lymphocytes and in their vicinity.

### Within MS lesions, elevated levels of secreted IL-16 correspond to increased CD4+ Th1 infiltration and signaling, as measured by T-bet expression, and Stat-1 phosphorylation

**Figure 3:**
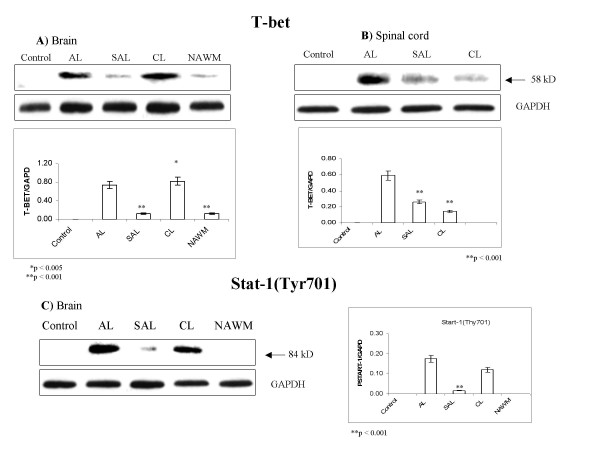
**Elevated levels of T-bet and phosphorylated Stat-1 suggest substantial infiltration by CD4+ Th1 cell in MS lesions**. Expression of T-bet was not detected in control brain (**A**) or spinal cord (**B**). In MS lesions, relatively high levels T-bet were measured in acute lesions (AL) and chronic lesions (CL) in brain, and in AL in spinal cord. Compared to AL, markedly lower levels of T-bet were found in subacute lesions (SAL) and NWAM. **C**) Regulation of Stat-1 was similar to that of T-bet in brain. Phospho-Stat-1 (Tyr^701^) was not detected in control brain. Conversely, it was abundant in AL and CL in brain. Representative blots are shown from a total of 5 experiments analyzing 39 MS cases and 19 controls. Relative levels of T-bet/GAPDH and Stat-1 (Tyr^701^)/GAPDH were estimated by densitometry. Mean values ± SD are shown in corresponding graphs.

T-bet was not detected in control brain and spinal cord, but T-bet levels were appreciable in MS lesions (Fig. 3). Levels of T-bet were markedly increased in acute and chronic MS lesions in brain, (Fig. 3A) and in acute spinal cord lesions (Fig. 3B), corresponding to increases in IL-16. Levels of T-bet in spinal cord were greatest in acute lesions, and gradually decreased in subacute and chronic lesions. This pattern of T-bet regulation was expected based on routine histopathology of these lesions, where inflammatory infiltration also decreased from acute to subacute and chronic lesions [5]. In brain lesions, levels of T-bet were almost equally high in acute and chronic lesions, and corresponded to similarly high levels of secreted IL-16 (Fig. 1A). Conversely, in spinal cord, T-bet and secreted IL-16 levels were decreased in subacute and chronic as compared to acute lesions (Fig. 1B and Fig. 3B).

Intrathecal levels of Stat-1 (Tyr ^701^) were undetectable in controls, but were found at appreciable levels in acute MS lesions, and at lower levels in subacute and chronic MS lesions in brain and spinal cord (Fig. 3C). Regulation of Stat-1 (Tyr ^701^) correlated well with trends measured for T-bet and secreted IL-16 in MS lesions. In contrast to increased phosphorylation of Stat-1, we did not detect phosphorylated Stat-6 (Tyr^641^) (not shown), suggesting that Stat-1-specific signaling is preferential over Stat-6-specific activation in MS lesions.

Extensive IL-16 immunostaining, confined to infiltrating cells, was observed in MS lesions. Less widespread immunostaining was found in normal-appearing white matter of brain and spinal cord. IL-16 immunoreactivity was readily observed in CD3+ T cells in perivenular infiltrates (Fig. 6A). Within the pool of infiltrating mononuclear cells, a subset of T-bet+ cells frequently expressed IL-16 (Fig. 4A). IL-16 nuclear immunostaining was seldom observed, while IL-16 often appeared as cytoplasmic or membrane-bound immunostaining, or adjacent to cells as secreted product. IL-16 was found frequently between adjoining mononuclear cells (Fig. 4A and 4B).

In addition to co-localization with CD3+ and CD4+ T cells, some IL-16 immunoreactivity was confined to CD8+ T cells, CD20+ B cells, and CD83+ dendritic cells (not shown). Occasional Mac-1+ microglia were IL-16+, while the majority of cells expressing Mac-1 did not contain IL-16 (not shown).

### Intrathecal production of IL-16 correlates with increased levels and fragmentation of phosphorylated neurofilament medium and heavy chains [NF(M+H)]P

Phosphorylated neurofilament medium (160 kD) and heavy (220 kD) chains [NF(M+H)]P, were detected in control brain and spinal cord tissue at appreciable levels.

**Figure 5:**
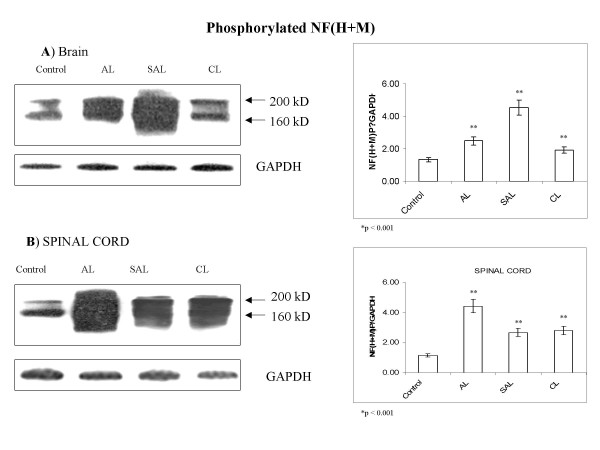
**Alterations of axonal cytoskeleton are suggested by markedly increased levels of phosphorylated medium and heavy chains of neurofilament [NF (M+H)P] in MS lesions**. Relative levels of NF (M+H)P are markedly increased in MS lesions in (**A**) brain and spinal (**B**) cord compared to control values. Control tissue displays two distinct bands corresponding to 160-kD medium and 200-kD heavy neurofilament chains. In contrast, MS lesions show bands that cannot be clearly distinguished due to hyperphosphorylation. Representative blots are shown from a total of 5 experiments analyzing 39 MS cases and 19 controls. Relative levels of NF(M+H)P/GAPDH are estimated by densitometry. Mean values ± SD are shown in corresponding graphs.

In MS lesions, levels of these neurofilaments were highly upregulated compared to control tissue. Upregulation of [NF(M+H)]P was readily accompanied by its degradation in MS lesions. The most prominent change in levels and degradation of [NF(M+H)]P was observed in acute lesions in brain and spinal cord (Fig. 5A and 5B). In subacute and chronic lesions, degradation of [NF(M+H)]P persisted; levels were approximately one half those of acute lesions but still markedly higher than those of controls. The observed patterns of regulation of [NF(M+H)]P correspond to those of IL-16 (Fig. 1) in MS lesions.

Abnormalities in appearance of phosphorylated neurofilaments (medium and heavy) chains [NF(M+H)]P, were observed in MS lesions and, interestingly, also in NAWM. Small, rounded, irregularly shaped fragments of neurofilaments were readily observed (Fig. 6B). IL-16 immunostaining was found either confined to mononuclear cells or in the proximity of cells adjacent to irregular appearing neurofilaments (Fig. 6B).

## Discussion

Our observations suggest an important role for the cytokine IL-16 in regulation of CD4+ T cell infiltration, of severity and frequency of relapses, and of subsequent demyelination and axonal damage, in a mouse model of EAE [19]. These observations suggest that IL-16, which serves as a specific ligand for the CD4 co-receptor, may have a similar role or roles in regulation of MS. In other organs, increased levels and processing of IL-16 is associated with the pathogenesis of delayed type hypersensitivity [23] and autoimmunity, such as in atopic dermatitis [24], with autoimmune rheumatoid arthritis and lupus [25,26]. However, the role of IL-16 regulation in MS lesions is largely unknown. With the exception of one report, which shows IL-16 immunoreactivity within lesions of a single case of MS, this has been little studied and the mechanisms of IL-16 regulation in MS remain elusive [27]. In this previous study, it was not apparent if IL-16 expression in MS differed from that of control adult CNS, or if the IL-16 expression was due to precursor or secreted IL-16. The cellular localization of IL-16 immunoreactivity suggested expression in microglia-like cells, although it did not correspond with the expression of several microglia activation markers. Some of the IL-16 immunoreactivity was found to be related to lymphocytes and astrocyte-like cells.

To asses the potential role of locally produced IL-16 in regulation of neuroinflammation and axonal damage, key markers of MS pathology, we specifically addressed the following questions: Does production of mature IL-16 occur within MS lesions? What are the phenotypes of IL-16-containing or -producing cells? Does intrathecal IL-16 accumulation correlate with specific CD4+ Th1 inflammation and damage of axonal cytoskeletons?

Here we present our findings on the expression and distinct regulation of pro- and secreted IL-16 in acute, subacute and chronic MS lesions, and in normal-appearing white matter, from brain and spinal cord of 39 patients with MS and 19 normal controls.

As anticipated, the highest levels of pro- and secreted IL-16 were found in acute lesions. The marked increases in secreted IL-16 over control levels in brain and spinal cord suggests a role for IL-16 in regulation of neuroinflammation in acute lesions. Locally produced IL-16 may further support production of inflammatory cytokines by infiltrating mononuclear cells, in a fashion similar to that seen *in vitro *[17]. In this way, IL-16 may support inflammation in a complex network of pro- and anti- inflammatory pathways occurring in acute lesions [28,29]. Surprisingly high levels of secreted IL-16 in chronic lesions, and lower but still above normal levels in normal appearing white matter, may be due to generalization of inflammatory and degenerative processes beyond focal demyelinating plaques and beyond macroscopically defined lesions. Such pathology is consistent with diffuse inflammation and with accumulating axonal injury during the progressive course of MS, as suggested by others [30]. Alternatively, IL-16 found in chronic lesions and in normal white and grey matter adjacent to lesions may originate from neuronal IL-16 (NIL-16). Lymphoid IL-16 is transcribed from an intronic promoter lying within the NIL-16 gene. C-terminal regions of IL-16 and NIL-16 share 100% similarity. NIL-16 is produced by neurons primarily in cerebellum and in hippocampus. In vitro, at least, neuronal apoptosis leads to cleavage of NIL-16 by active caspase-3 and secretion of C-terminal bioactive IL-16 [31]. Whether similar mechanisms operate *in vivo *during neuronal damage is not known. The lack of NIL-16 in normal control samples may reflect the anatomic distribution of NIL-16 expression. Alternatively, epitope for mAb 14.1 may be masked in NIL-16. Even if this antibody does detect intact NIL-16, following cleavage with active caspase-3, it would not detect the N-terminal domain of NIL-16. Our finding of IL-16 in MS tissue devoid of macroscopically significant mononuclear infiltration and/or microglial activation argues for a role for IL-16 in progression and spreading of focal inflammatory lesions. Our data showing substantially increased secreted IL-16 in CNS of mice with chronic EAE [20] support this proposed role for IL-16.

Slight differences were observed between brain and spinal cord in regulation of IL-16, active caspase-3, T-bet, Stat-2 (Tyr^701^) and NF(M+H)P, which may suggest the existence of distinct regulatory mechanisms of neuroinflammation in these two regions. The absence of appreciable IL-16 expression in control brain and spinal cord argues against an important physiological role for IL-16 in the maintenance of homeostasis within normal CNS.

In MS lesions, putative producers of IL-16 include CD4+, CD8+ T cells, B cells, dendritic cells, and subsets of activated microglia. Locally produced bioactive IL-16 may exert its paracrine effects by binding to CD4 co-receptor. A minimal peptide RRKS, between Arg^106 ^and Ser^109 ^of C-terminally cleaved bioactive IL-16, is essential for the binding and signaling through CD4 [32]. This specific localization of intrathecal IL-16 bears relevance to its several potentially important roles: in local immunomodulation through regulation of CD4+T-T cell communication; in CD4+T cell helper function in the processes of T-B cell communication; and lastly in local antigen presentation by dendritic cells and microglia. A role for IL-16 in the cross talk between dendritic cells and T cells has also been proposed [33].

The Stat-1 protein plays a central role in the biological activity of both type I and type II interferons. Th1-derived IFNγ binds to its specific receptor on the surface of T cells and initiates signaling cascade, which includes Stat-1 phosphorylation and nuclear translocation. T-bet is regulated by IFNγ-, Stat-1-mediated signaling, and its level is markedly reduced in IFNγ- or Stat-1-mutant cells [34].

Inflammation-induced changes in axonal cytoskeletons, which include increased levels of phosphorylated medium and heavy chains of neurofilament, readily lead to axonal dysfunction [1,35]. Our finding of IL-16 immunoreactivity in the vicinity of degenerate neurofilaments, which appears to be secreted from mononuclear cells (Fig. 6), suggests that this proinflammatory cytokine may participate in damage to axonal cytoskeletons.

## Conclusion

We find extensive production of IL-16 in MS lesions and in adjacent normal-appearing white (NAWM) and grey matter (NAGM) in both brain and spinal cord of MS patients. Increased levels of secreted IL-16 correlated with elevated levels of active caspase-3, T-bet and Stat-1 (Tyr^701^). Within both MS lesions and NAWM, active-caspase-3+ IL-16+, and T-bet+IL-16+ mononuclear cells were readily observed. A specific localization of IL-16 to sites of cell-cell contact was frequently observed, suggesting a role in cell-to-cell communication. We found similarities in patterns of regulation between secreted IL-16 and active caspase-3 that suggest production of secreted IL-16 within lesions by active caspase-3-dependent cleavage of pro-IL-16. Conversely, similarities in patterns of regulation between IL-16, T-bet and Stat-1 (Tyr^701^) suggest contributions of CD4+Th1 cell subsets to IL-16 local production, and/or a role for locally produced secreted IL-16 in CD4+Th1 cell homing.

Increased production of secreted IL-16 corresponded with marked elevations in phosphorylated neurofilament medium and heavy chains, suggesting inflammation-induced changes in axonal cytoskeleton in MS lesions and NAWM. IL-16+ mononuclear cells were observed adjacent to these axons; these were frequent in MS lesions and sparse in NAWM.

Taken together, our data suggest an important role for intrathecally secreted IL-16 in specific regulation of CD4+Th1 cell homing, Th1-mediated neuroinflammation, and subsequent changes in axonal cytoskeleton in MS. Our results also suggest a role for IL-16 in processes of cell-to-cell communication in MS lesions.

## Abbreviations

IL-16: interleukin 16

EAE: experimental autoimmune encephalomyelitis

MS: multiple sclerosis

AL: acute lesion

SAL: subacute lesion

CL: chronic lesion

NAWM: normal appearing white matter

NAGM: normal appearing grey matter

NCWM: normal control white matter

NCGM: normal control grey matter

NF(M+H)P: phosphorylated medium and heavy chains of neurofilament

Stat-1: signal transducer and activator of transcription-1

## Competing interests

The author(s) declare that they have no competing interests.

## Authors' contributions

DSS conceived the design of the study, provided critical analysis of the data, and wrote the manuscript. She performed immunocytochemistry and confocal analysis of MS tissue sections. JC performed the western blot analysis and helped with immunostaining and statistical analysis. WWC provided critical analysis of the manuscript. JG selected and provided MS samples and performed routine histopathology analysis of MS lesions. He contributed to critical review of the manuscript. All authors read and approved the final version of the manuscript.
